# Supplementary material for: pH-Responsive Graphene Oxide-Based 2D/3D Composite for Enhancing Anti-Corrosion Properties of Epoxy Coating
Source: Nanomaterials (Basel). 2024 Feb 6;14(4):323. doi: 10.3390/nano14040323 (PMC10893030; doi:10.3390/nano14040323)
Supplement: Supplementary file 1 [file nanomaterials-14-00323-s001.zip › nanomaterials-2822086-supplementary.pdf]

nanomaterials

Article

pH−Responsive Graphene Oxide−Based 2D/3D Composite for Enhancing Anti−Corrosion Properties of Epoxy Coating

Jian Wang, Yangyang Cao, Jieru Wan, Meng Zhang, Yunqiang Li, Yanli Wang *, Dalei Song, Tao Zhang and Jun Wang

Key Laboratory of Superlight Materials and Surface Technology, Ministry of Education, College of Materials Science and Chemical Engineering, Harbin Engineering University, Harbin 150001, China; wjian@hrbeu.edu.cn (J.W.); cyyheu@hrbeu.edu.cn (Y.C.); wanjieru@hrbeu.edu.cn (J.W.); 2016101528@hrbeu.edu.cn (M.Z.); lyq17861821305@hrben.edu.cn (Y.L.); songdalei@hrbeu.edu.cn (D.S.); zhangtao@mail.neu.edu.cn (T.Z.); junwang@hrbeu.edu.cn (J.W.)

* Correspondence: yanliwang@hrbeu.edu.cn

Table S1. Fitted parameters of the EIS results and inhibition efficiency of different scratch coatings after 72 h of immersion.

Samples	C_c ($\Omega^{-1}.cm^{-2}$)	C_{dl} ($\Omega^{-1}.cm^{-2}$)	R_c ($\Omega.cm^2$)	R_{ct} ($\Omega.cm^2$)	Chi-square ($\chi 2$)	IE (%)
S-EP	1.76×10^{-6}	1.98×10^{-6}	8599	1.18×10^{4}	2.05×10^{-3}	
S-ZIF-90/GO-EP	4.09×10^{-5}	5.10×10^{-6}	18450	2.90×10^{4}	3.59×10^{-4}	59.3
S-ZIF-90-AAP/GO-EP	8.38×10^{-6}	3.67×10^{-5}	7100	1.11×10^{5}	8.10×10^{-3}	89.4

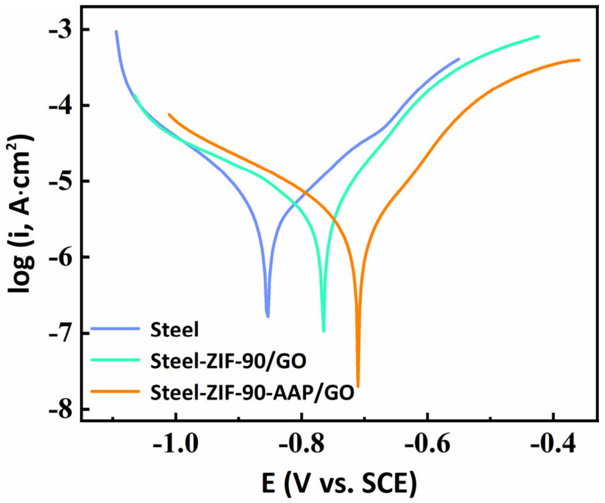

Figure S1. Potentiodynamic polarization curves of Steel, Steel-ZIF-90/GO and Steel-ZIF-90-AAP/GO.

Table S2. Electrochemical parameters extracted from polarization curves of the different steels after 72 h of immersion. .

Samples	E_{corr} (V)	i_{corr} ($A\cdot cm^{-2}$)	η (%)
Steel	-0.854	5.717×10^{-6}	-
Steel-ZIF-90/GO	-0.766	3.092×10^{-6}	45.9
Steel-ZIF-90-AAP/GO	-0.710	1.598×10^{-6}	72.1
